# Screening lactic acid bacteria and yeast strains for soybean paste fermentation in northeast of China

**DOI:** 10.1002/fsn3.3372

**Published:** 2023-07-19

**Authors:** Siyi Li, Linjie Guo, Jinhong Gu, Guangqing Mu, Yanfeng Tuo

**Affiliations:** ^1^ School of Food Science and Technology Dalian Polytechnic University Dalian China; ^2^ Dalian Probiotics Function Research Key Laboratory Dalian Polytechnic University Dalian China

**Keywords:** lactic acid bacteria, physicochemical property, safety, Soybean paste, volatile compound, yeast

## Abstract

Soybean paste was a traditional fermented product in northeast China, mainly fermented by molds, yeast, *Bacillus*, and lactic acid bacteria. In this study, the safety and fermentation ability of lactic acid bacteria and yeast strains isolated from traditional soybean paste in northeast China were evaluated, and the dynamic changes of biogenic amines, aflatoxin, total acids, amino acid nitrogen, and volatile compounds were investigated during the fermentation of the traditional soybean paste. Among the tested strains, *Lactiplantibacillus plantarum* DPUL‐J8 could decompose putrescine by 100%, and no biogenic amine was produced by *Pichia kudriavzevii* DPUY‐J8. *Lactiplantibacillus plantarum* DPUL‐J8 and *P. kudriavzevii* DPUY‐J8 with strong biogenic amine degrading capacities were inoculated into the soybean paste. After 30 days of fermentation, the content of biogenic amines and aflatoxin in the fermented soybean paste declined by more than 60% and 50%, respectively. At the same time, compared with the control group without inoculation, the contents of total acid (1.29 ± 0.05 g/100 g), amino acid nitrogen (0.82 ± 0.01 g/100 g), and volatile compounds in soybean paste fermented by *L. plantarum* DPUL‐J8 and *P. kudriavzevii* DPUY‐J8 were significantly increased, which had a good flavor. These results indicated that the use of *L. plantarum* DPUL‐J8 and *P. kudriavzevii* DPUY‐J8 as starter cultures for soybean paste might be a good strategy to improve the safety and flavor of traditional Chinese soybean paste.

## Graphical abstract

In this study, *L. plantarum* DPUY‐J8 and P. kudriavzevii DPUY‐J8 were used as starter cultures of soybean paste, which could significantly increase the contents of total acids, amino acid nitrogen, and volatile compounds in fermented soybean paste. After simultaneous fermentation for 30 d, the contents of biogenic amines and aflatoxins in fermented soybean paste decreased by more than 60% and 50%, respectively. Therefore, using *L. plantarum* DPUY‐J8 and *P. kudriavzevii* DPUY‐J8 as starter cultures of soybean paste may be a good strategy to improve the safety and flavor of traditional soybean paste.

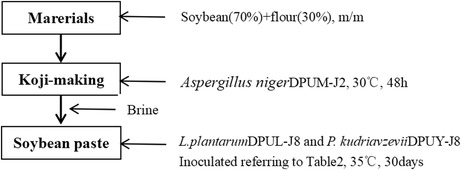

## 1 INTRODUCTION

Soybean paste has a long history of manufacture and consumption in northeast China. Soybean paste is mainly made of soybeans, and the nutritional components of soybeans mainly include protein, fat, and calcium. In cereals and other leguminous plants, the protein content of soybean is the highest, at about 40%, and there are also phospholipids, vitamins, and minerals in soybean. In addition, there are phytic acid, oligosaccharides, isoflavones, and other substances in soybeans (Tang et al., 2013). Due to its good flavor and unique taste, soybean paste is popular all over the world (Jia et al., 2018; Kuligowski et al., 2017; Papageorgiou et al., 2018). The making process of soybean paste is mainly divided into three stages: the treatment of raw materials, the preparation of koji, and the fermentation of soybean paste (Chettri & Tamang, 2015; Li, Rui, et al., 2017; Li, Zhao, et al., 2017). The traditional fermentation of soybean paste takes a long time to achieve the desired flavor, and at the same time has a high risk of contamination with undesired microbes and toxins (Guan et al., 2013; Jia et al., 2018).

The unique flavor of soybean paste is attributed to microbes involved in fermentation and heir metabolites (Cui et al., 2015; Jung et al., 2016). In the process of soybean paste fermentation, the ingredients of raw materials are decomposed and recombined under the action of various microbial enzymes to produce various active substances, such as functional peptides generated by protein degradation, furanones, and melanoids generated by the Maillard reaction between amino groups in protein decomposition products and carbonyl groups in reducing sugars. At the same time, various bioactive substances interact with each other to form the unique physiological functions of fermented soybean food, such as promoting digestion, regulating intestinal flora, and having antioxidant effects (Xu, 2018). Lactic acid bacteria (LAB) and yeast are the dominant microorganisms in traditional soybean paste, playing an important role in fermented soybean foods (Li, Rui, et al., 2017; Li, Zhao, et al., 2017). Yeast is capable of alcoholic fermentation and the hydrolysis of various amino acids into their respective alcohols, leading to the accumulation of alcoholic substances that give the soybean paste its unique flavor (Wah et al., 2013; Yokotsuka, 1986). The main contributor to the safety and flavor of fermented products is LAB, which produces acetic acid and lactic acid (Di Cagno et al., 2013; Uchida et al., 2005). However, microbial metabolism may give rise to potentially harmful substances such as biogenic amines (Liang et al., 2019; Shukla et al., 2010, 2014). Biogenic amines are low‐molecular‐weight nitrogen compounds produced by microbial decarboxylation of amino acids and nitrogen compounds during soybean paste fermentation, and ingestion of high concentrations of biogenic amines may cause severe health problems (Chun et al., 2020; Jung et al., 2016; Kim & Ji, 2015; Moon et al., 2010). It is possible to improve the safety and quality of soybean paste by applying the appropriate starter cultures to the soybean paste fermentation (Wah et al., 2013; Yang et al., 2019). Zhao et al. (2020) used *Staphylococcus carnosus* M43 *and Pediococcus acidilactici* M28 as the starter cultures to ferment soybean paste, which not only reduced the generation of biogenic amines but also produced more ideal flavor compounds. Cui et al. (2015) reported that the co‐culture of halophilic LAB (*Tetragenococcus halophilus*) and yeasts (*Zygosaccharomyces rouxii* and *Candida verslis*) was conducive to the improvement of soy sauce flavor during the fermentation. Zhang et al. (2012) reported that adding a compound starter culture including *Lactiplantibacillus plantarum*, *Saccharomyces cerevisiae*, and *Candida* to broad bean paste not only maintained the traditional characteristics but also reduced the aflatoxin B1 content and improved the aroma components compared with the traditional process, and the production cycle was shortened by 1/3.

In this study, the safety of yeast and LAB strains isolated from the traditional fermented soybean paste in northeast China was evaluated in terms of biogenic amines, hemolysis, and antibiotic sensitivity. Furthermore, the protease activity of the strains was assessed. The selected strains with safety and protease activity were used as starter cultures for the soybean paste fermentation to explore the effects of the strains on the main physicochemical properties and volatile compounds during the fermentation of soybean paste.

## 2 MATERIALS AND METHODS

### 2.1 Bacterial strains and culture conditions

In this study, 16 strains of LAB and 15 strains of yeast, which were isolated from traditional soybean paste samples in our previous work (Li et al., 2021), were subjected to safety and protease assessments. Lactic acid bacteria were cultured in MRS medium at 37°C, and yeast was cultured in YPD medium at 28°C. *Aspergillus niger* DPUM‐J2 for koji preparation was preserved in the Dalian Probiotics Function Research Key Laboratory, Dalian Polytechnic University.

### 2.2 Biogenic amines producing ability of the LAB and yeast strains

The ability of the strains to produce biogenic amines was determined according to the method by Li et al. (2021). Eight kinds of 0.1% precursor amino acids and 0.005% pyridoxal phosphate were added to the MRS and YPD liquid culture media. LAB strains were inoculated into the above MRS medium and cultured at 37°C for 48 h. Yeast were inoculated into the above YPD at 28°C for 48 h. Dansyl chloride was used for derivatization. The content of biogenic amine was detected by a high‐performance liquid chromatography system (Huapu, S6000), monitored by a UV detector at 235 nm, and a Tnature C18 column (5 μm, 4.6 × 250 mm, Waters). Water was used as mobile phase A, acetonitrile was used as mobile phase B, and the gradient elution procedure is shown in Table 1. The flow rate was 1.0 mL/min, the column temperature was 40°C, and the injection volume was 30 μL.

**TABLE 1:** Elution gradient.

Elution time/min	Mobile phase/%	Mobile phase/%
0	45	55
10	45	55
15	35	65
20	20	80
25	20	80
30	10	90
33	10	90
35	45	55

### 2.3 Screening strains with biogenic amine decomposing ability

The ability of LAB to break down biogenic amines was measured using a method by Eom et al. (2015) with a few small changes. MRS liquid medium containing 1‰ eight biogenic amine standards and a pH value of 5.5 was used to screen strains for biogenic amine decomposing ability. The LAB were inoculated into the MRS medium containing biogenic amine and cultured at 37°C for 48 h. A MRS medium containing biogenic amine without microorganism inoculation was used as the control. The culture medium was centrifuged (2800 *g* for 10 min) to obtain the supernatant. The biogenic amine content in the supernatant was analyzed by HPLC to determine the biogenic amine degrading ability of the strain.

### 2.4 Hemolysis verification of strains

The sterilized Columbia medium (Hopebio) was added with 5% sheep blood (Solarbio Beijing) in an ultraclean bench and shaken evenly. The LAB and yeast strains were streaked on the Columbia agar medium and incubated at 37°C for 48 h to observe whether there was a clear circle around the strain colony as described previously (Jeong et al., 2016). The presence of a grass‐green ring was considered as α hemolytic; the presence of a clear hemolytic ring was considered as β hemolysis; and the absence of change was considered non‐hemolysis. *Listeria monocytogenes* was used as a positive control for hemolysis analysis.

### 2.5 Detection of protease activity of strains

About 5 g soybean and 50 mL of 2.5 mol/L NaCl solution were added into a conical flask, and sterilized at 121°C for 20 min to prepare fermentation medium. The LAB and yeast strains to be tested were inoculated into the fermentation medium at 2% (v/v) and cultured at 37 and 28°C for 48 h. The culture samples were centrifuged (11100 *g* for 10 min) to obtain the supernatant as a crude enzyme solution for determining protease activity. The protease activity of the crude enzyme solution was determined by a protease assay kit (YBE‐1819) (YBio).

### 2.6 Antibiotic sensitivity test of strains

The drug resistance of the strains was detected by the method described by Shi et al. (2020) with some modifications. Nine antibiotics, including ampicillin, chloramphenicol, kanamycin, gentamicin, penicillin, tetracycline, vancomycin, clindamycin, and levofloxacin, were selected from the list of commonly used antibiotics for the drug sensitivity test for LAB. Seven antibiotics, including econazole, itraconazole, clotrimazole, ketoconazole, miconazole, amphotericin, and fluconazole, were selected for the yeast drug sensitivity test. The RCA and YPD media were prepared, and after sterilization, the strains to be tested were poured on a flat plate. Antibiotic discs were put on RCA and YPD agar, and then cultured at 37°C for 48 h. The diameter of the bacteriostatic circle was used to distinguish sensitivity to antibiotics. Identification of antibiotic susceptibility by inhibition zone diameter: S, sensitive (≥20 mm); I, intermediate sensitive (10–20 mm); R, resistant (≤10 mm).

### 2.7 Identification of LAB and yeast strains

The strains with BA‐degradation ability and high protease activity were identified through 16S rDNA and 26S rRNA sequencing analysis. The primers for LAB strains PCR amplification were 338F (ACTCCTACGGGAGGCAGCAG) and 806R (GGACTACHVGGGTWTCTAAT). The fungal gene design primers ITS1F (CTTGGTCATTTAGAGGAAGTAA) and ITS2R (GCTGCGTTCTTCATCGATGC) were amplified by PCR and analyzed for 26 srRNA sequence. The amplified 16S rDNA and 26S rRNA gene product sequence comparison was carried out with the available database using BLAST analysis in NCBI server.

### 2.8 Preparation of soybean paste

**FIGURE 1:**
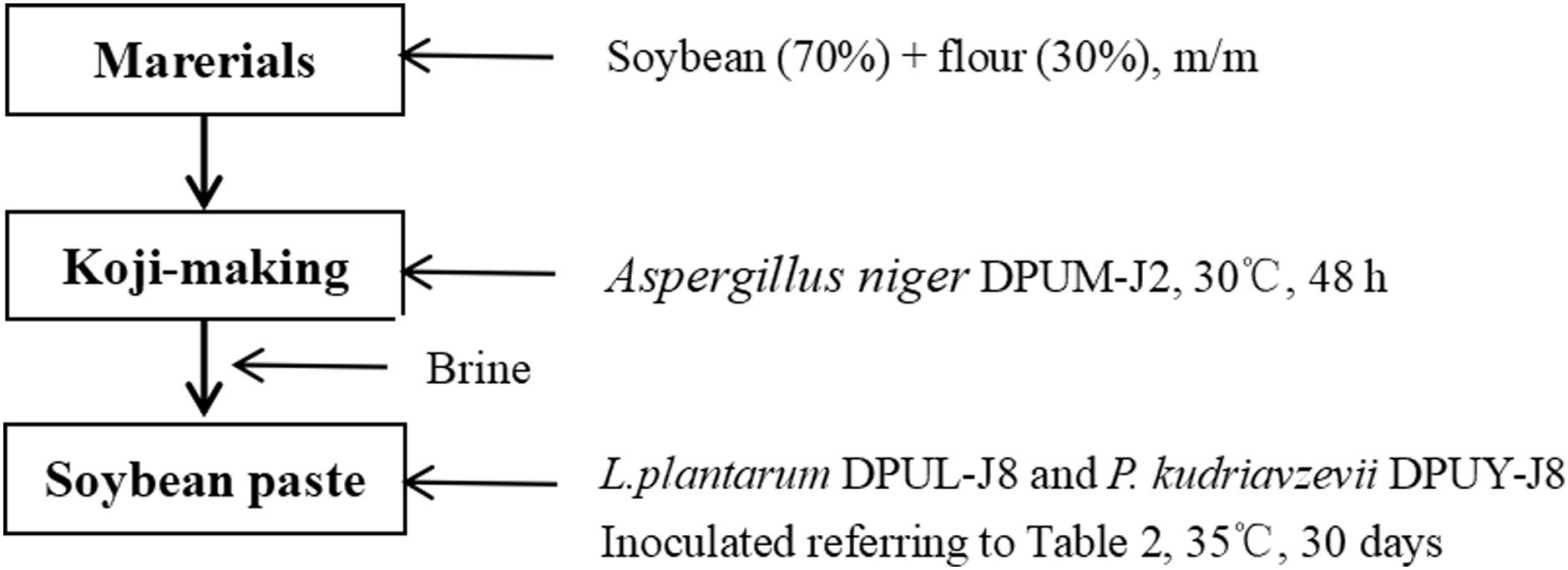
Manufacture procedure of soybean paste samples.

Soybean paste is a traditional Chinese fermented food. In this study, soybean paste was made according to the traditional method, as shown in Figure 1 and Table 2. Firstly, the *Aspergillus niger* DPUM‐J2 was inoculated into PDA medium and cultured at 28°C for 72 h to obtain cultures containing spores, which were stored in the refrigerator at 4°C for later use. The raw soybeans were soaked in water for 4 h and steam cooked at 121°C for 20 min. Soybean and flour were mixed at a ratio of 7:3 and cooled to 35°C. *A. niger* DPUM‐J2 spore suspension was added to the soybean and flour mixture and incubated for 48 h at 30°C for koji making, until the surface of the mixture was coated with *A. niger* DPUM‐J2 mycelium. The prepared koji was added to the same mass brine (10%, w/v) and divided into four groups: control, DPUL‐J8, DPUY‐J8, and DPUL‐J8+DPUY‐J8, then fermented at 35°C for 30 days. Different groups were inoculated with different strains, just as described in Table 2. The control group was not inoculated with *L. plantarum* DPUL‐J8 and *P. kudriavzevii* DPUY‐J8 and used as a blank. DPUL‐J8 group was inoculated with *L. plantarum* DPUL‐J8, DPUY‐J8 group was inoculated with *P. kudriavzevii* DPUY‐J8, and DPUL‐J8+DPUY‐J8 group was co‐inoculated with *L. plantarum* DPUL‐J8 and *P. kudriavzevii* DPUY‐J8. Fermented soybean samples were taken in each group every 5 days during the fermentation, and all samples were maintained at −80°C for later analysis.

**TABLE 2:** Fermentation parameters of soybean paste samples.

Samples	Koji‐making	Brine		Soybean paste fermentation	
	Soybean/flour (m/m)	Concentration (%)	Koji/brine (m/m)	Temperature (°C)	Strains
Control	7:3	10	1:1	35	‐
DPUL‐J8	7:3	10	1:1	35	*Lactiplantibacillus plantarum* DPUL‐J8
DPUY‐J8	7:3	10	1:1	35	*Pichia kudriavzevii* DPUY‐J8
DPUL‐J8+DPUY‐J8	7:3	10	1:1	35	*L. plantarum* DPUL‐J8, *P. kudriavzevii* DPUY‐J8

*Note*: “‐”: no strain added.

### 2.9 Measurement of biogenic amines, aflatoxin B1 and physicochemical parameters in the fermented soybean paste samples

The physicochemical parameters of the soybean paste samples at different fermentation periods were measured according to the Chinese national standard GB/T24399‐2009.

Analysis of biogenic amines in soybean paste samples was carried out based on the methods described by Jia et al. (2018). A quantity of 5 g of soybean paste sample was weighed and placed in a 100 mL beaker accurately. A volume of 20 mL of 5% trichloroacetic acid was added, stirred for 60 min, and centrifuged. The operation was conducted continuously for two times, and the supernatant was collected at a constant volume and filtered through filter paper, and 10 mL of n‐hexane was added. After being shaken and mixed thoroughly, the upper layer was sucked out, and the lower layer liquid was collected. Two consecutive operations were conducted for standby. Derivatization and chromatographic condition were the same as those described in Section 2.2.

Aflatoxin B1 content in the soybean paste samples was determined according to the instructions of the aflatoxin B1 detection kit YB‐9601B1 (YBio). About 5 g of the ground soybean paste sample was added to 10 mL of a 70% methanol solution, shaken vigorously for 3 min, then filtered, and the filtrate was collected for later use.

The pH value of soybean paste samples was detected by a pH meter. The determination of total acids in soybean paste samples was analyzed by NaOH titration (Kim et al., 2018). 40 mL of water was added to 5 g soybean paste without particles, and transferred to a 50 mL volumetric flask for constant volume. 30 mL of deionized water was added to the 10 mL of the above liquid, its pH was titrated to 8.2 with NaOH, the consumed volume of sodium hydroxide was recorded, and 40 mL of deionized water was used as a blank to repeat the above steps.

3, 5‐Dinitrosalicylic acid method was used to determine the content of reducing sugar during the fermentation of soybean paste (Kim & Lee, 2008).

Determination of amino acid nitrogen in soybean paste samples was analyzed by formol titration (Xie et al., 2018). 10 mL of a 36% solution of formaldehyde was added to the above solution, and the solution was titrated to pH 9.2 with sodium hydroxide solution while 40 mL of deionized water was used as the blank, and the above procedure was repeated.

### 2.10 Determination of volatile flavor compounds

The volatile flavor compounds in soybean paste samples were measured by GC–MS as described by Jeong et al. (2019). Soybean paste sample (3.0 g) was mixed with 10 μL cyclohexanone [10 μg/mL (w/v) in ethanol] as an internal standard in 20 mL SPME vials. The sample bottle was put in a water bath at 65°C for 30 min, and the volatiles were absorbed by the 50/30 μm DVB/CAR/PDMS SPME fiber (Supelco). Volatile compounds were analyzed using a gas chromatograph (7890A) equipped with a mass selective detector (5975C) (Agilent). Helium as a carrier gas at a flow rate of 1.0 mL/min. The oven temperature procedure was as follows: 35°C for 3 min, 3°C/min to 50°C, 6–150°C, 10–230°C, and 230°C for 6 min. The other conditions were a collection mass range of 40–350 m/z, an ion source temperature of 230°C, and an electronic ionization (EI) mode of 70 eV. Volatile fractions were searched in NIST. Quantitative analysis was performed according to the concentration of the internal standard and the ratio of the peak areas of each component in the sample to the peak areas of the internal standard.

### 2.11 Statistical analysis

All experiments included the determination of the protease activity of the bacteria and yeast, biogenic amines, aflatoxin B1, pH, total acids, amino nitrogen, and flavor compounds were repeated three times. SPSS was used to evaluate the one‐way analysis of variance to verify significant differences between samples. When *p* < .05, the result is considered significant. Origin 8.5 drawing is selected for the drawing.

## 3 RESULTS AND DISCUSSION

### 3.1 Biogenic amines produced by the strains isolated from traditional northeast soybean paste

Excessive biogenic amines in fermented foods can do harm to human health. A total of 16 LAB and 15 yeast strains isolated from traditional northeast soybean paste were tested for biogenic amine production. As shown in Table 3, all of the LAB strains except strains K14 2 and K16 produced biogenic amines in MRS medium containing amino acids, with the biogenic amine content ranging from 1.19 ± 0.01 to 13.58 ± 0.22 mg/kg. Putrescine and tryptamine contents were relatively high, and phenylethylamine and tyramine were not detected in all strains. The biogenic amine content produced by strain K17 reached to 13.58 ± 0.22 mg/kg, which was significantly higher than that of other strains (*p* < .05). As shown in Table 4, the yeast strains except for K11 and M1 did not produce biogenic amine in the medium containing amino acids. It was reported that the *Tetragenococcus halophilus* strain (MJ4) isolated from fish sauce did not produce BAs and was used as a starter culture for repressing biogenic amine formation in salted shrimp fermentation (Kim et al., 2019). The strains producing no or low levels of biogenic amines were considered safe and can be used for the soybean paste fermentation.

**TABLE 3:** Biogenic amine content produced by lactic acid bacteria (mg/kg).

Strains	Put	His	Try	Cad	Phe	Tyr	Spd	Spm	BAs
Blank	0.47 ± 0.16^k^	ND	0.91 ± 0.06	ND	ND	ND	ND	ND	1.38 ± 0.22^m^
K3	1.61 ± 0.03^e^	ND	ND	ND	ND	ND	ND	ND	1.61 ± 0.03^fg^
M2‐2	1.87 ± 0.01^c^	0.25 ± 0.02^c^	ND	ND	ND	ND	ND	ND	2.12 ± 0.04^e^
K14	1.03 ± 0.03^g^	0.16 ± 0.01^d^	ND	ND	ND	ND	ND	ND	1.19 ± 0.01^g^
O10	1.49 ± 0.24^f^	0.06 ± 0.04^e^	ND	ND	ND	ND	ND	ND	1.55 ± 0.28^fg^
M16	1.81 ± 0.02^d^	0.27 ± 0.07^b^	ND	ND	ND	ND	ND	ND	2.08 ± 0.10^e^
M44	2.21 ± 0.05^b^	ND	5.66 ± 0.14^b^	ND	ND	ND	ND	ND	7.87 ± 0.20^c^
K4	2.63 ± 0.14^a^	ND	6.78 ± 0.38^a^	ND	ND	ND	ND	ND	9.41 ± 0.52^b^
K6	1.20 ± 0.06 ^h^	0.94 ± 0.03^a^	ND	ND	ND	ND	ND	ND	2.15 ± 0.10^e^
D43	1.34 ± 0.01^i^	ND	ND	ND	ND	ND	ND	ND	1.34 ± 0.01^g^
M43	ND	ND	2.22 ± 0.04^d^	ND	ND	ND	ND	ND	2.22 ± 0.04^e^
K17	ND	ND	2.22 ± 0.06^d^	1.83 ± 0.02^b^	ND	ND	9.16 ± 0.11	0.35 ± 0.02	13.58 ± 0.22^a^
D2‐6	ND	ND	5.05 ± 0.49^c^	ND	ND	ND	ND	ND	5.05 ± 0.49^d^
WZ	ND	ND	ND	1.49 ± 0.29^c^	ND	ND	ND	ND	1.49 ± 0.29^fg^
K9	ND	ND	ND	1.86 ± 0.01^a^	ND	ND	ND	ND	1.86 ± 0.01^ef^
K14 2	ND	ND	ND	ND	ND	ND	ND	ND	ND
K16	ND	ND	ND	ND	ND	ND	ND	ND	ND

*Note*: Values are expressed as averages of three independent experiments ±SD. ND indicates that no biogenic amines have been detected (*p* < .05).

**TABLE 4:** Biogenic amine content of yeast (mg/kg).

Strains	Put	His	Try	Cad	Phe	Tyr	Spd	Spm	BAs
K11	ND	2.80 ± 0.10	ND	ND	ND	ND	ND	ND	2.80 ± 0.10
K8	ND	ND	ND	ND	ND	ND	ND	ND	ND
K10	ND	ND	ND	ND	ND	ND	ND	ND	ND
M4‐1	ND	ND	ND	ND	ND	ND	ND	ND	ND
K1	ND	ND	ND	ND	ND	ND	ND	ND	ND
D4‐1	ND	ND	ND	ND	ND	ND	ND	ND	ND
K5	ND	ND	ND	ND	ND	ND	ND	ND	ND
M5	ND	ND	ND	ND	ND	ND	ND	ND	ND
M1	ND	ND	ND	1.10 ± 0.12	ND	ND	ND	ND	1.10 ± 0.12
D4‐3	ND	ND	ND	ND	ND	ND	ND	ND	ND
M4‐2	ND	ND	ND	ND	ND	ND	ND	ND	ND
O2‐1	ND	ND	ND	ND	ND	ND	ND	ND	ND
K13	ND	ND	ND	ND	ND	ND	ND	ND	ND
M2	ND	ND	ND	ND	ND	ND	ND	ND	ND
O2‐6	ND	ND	ND	ND	ND	ND	ND	ND	ND

*Note*: Values are expressed as averages of three independent experiments ±SD. ND indicates that no biogenic amines have been detected.

### 3.2 Screening of biogenic amine degrading strains

Furthermore, the biogenic amine content in the blank control group was 1.38 ± 0.22 mg/kg, and the biogenic amine content of the MRS medium inoculated with some strains was lower than that of the blank control group, indicating that the strains might degrade biogenic amines. Sixteen LAB were further tested for their ability to degrade biogenic amines. As can be seen from Table 5, compared with the control group, the degradation rates of spermidine by strains K14 2 and K9 were above 80%, the degradation rates of putrescine by strains D43 and K14 2 were above 100%, and the degradation rates of cademine by strains D43, K14 2 and K9 were above 90%. The biodegradability of strain K14 2 to phenethylamine was 71.62 ± 0.06%, and the biodegradability of these strains was significantly higher than that of other strains (*p* < .05). HIS and TYR have the highest BAs toxicity in soybean paste, so the degradation of HIS and TYR is crucial for the safety of soybean paste (Zhao et al., 2020). The degradation rates of histamine and tyramine of strains D43, K14 2, and K9 were all over 60%, which showed significant bioamine degradation ability compared with other lactic acid bacteria.

**TABLE 5:** Degradation of biogenic amine by lactic acid bacteria (%).

Strains	Put	His	Try	Cad	Phe	Tyr	Spd
K3	46.41 ± 0.03^i^	58.24 ± 0.09^h^	48.09 ± 0.09^n^	51.57 ± 0.04^i^	51.94 ± 0.09^l^	62.45 ± 0.03^h^	13.65 ± 0.58^l^
K14	56.80 ± 2.21^f^	68.45 ± 0.36^d^	52.79 ± 0.37^k^	57.84 ± 0.33^f^	56.61 ± 0.40^i^	57.44 ± 1.45^j^	17.63 ± 0.72^l^
M2‐2	51.40 ± 0.45^g^	68.86 ± 0.14^d^	61.35 ± 0.26^f^	56.50 ± 0.52^g^	63.80 ± 0.05^e^	74.82 ± 0.28^c^	52.35 ± 1.00^f^
O10	49.61 ± 0.79^h^	61.33 ± 0.99^g^	60.70 ± 0.46^g^	51.44 ± 1.23^i^	62.16 ± 0.04^f^	73.07 ± 0.53^d^	50.19 ± 4.89^g^
M16	87.07 ± 0.63^c^	53.32 ± 0.19^j^	49.46 ± 0.24^m^	83.96 ± 0.72^e^	51.41 ± 0.19^l^	79.03 ± 1.01^a^	28.00 ± 3.10^k^
M44	60.19 ± 0.69^e^	50.45 ± 0.11^k^	53.15 ± 0.14^j^	48.77 ± 0.79^k^	55.73 ± 0.12^j^	48.49 ± 0.37^k^	53.00 ± 0.44^e^
K4	49.42 ± 0.98^h^	45.56 ± 0.85^l^	50.27 ± 0.31^l^	49.85 ± 0.76^j^	51.03 ± 0.32^l^	67.85 ± 0.66^g^	42.59 ± 0.55^h^
K6	42.90 ± 0.43^j^	63.73 ± 0.05^f^	75.08 ± 0.03^b^	46.90 ± 0.42^m^	58.14 ± 0.06^g^	71.45 ± 0.35^e^	19.94 ± 1.5^l^
D43	100^a^	78.91 ± 0.43^a^	71.88 ± 0.22^c^	98.37 ± 0.90^a^	74.75 ± 0.18^a^	68.33 ± 0.63^f^	79.67 ± 0.20^c^
M43	41.34 ± 1.06^k^	61.24 ± 0.36^g^	54.37 ± 0.56^i^	46.91 ± 1.01^m^	57.86 ± 0.56^h^	68.72 ± 0.61^f^	ND
K17	49.41 ± 0.25^h^	50.92 ± 0.08^k^	55.95 ± 0.11^h^	49.23 ± 1.37^j^	55.60 ± 0.08^j^	52.86 ± 0.37^j^	35.53 ± 0.61^j^
D2‐6	70.44 ± 0.82^d^	74.80 ± 0.15^b^	76.49 ± 0.07^a^	84.28 ± 0.16^d^	72.56 ± 0.17^b^	76.43 ± 2.18^b^	77.44 ± 1.54^d^
WZ	46.39 ± 0.55^i^	54.34 ± 0.54^i^	53.15 ± 0.14^j^	47.46 ± 0.91^l^	57.89 ± 0.02^h^	47.99 ± 0.04^l^	35.39 ± 0.01^j^
K9	96.78 ± 0.11^b^	58.93 ± 0.08^h^	65.46 ± 0.05^e^	94.57 ± 0.11^c^	65.14 ± 0.05^d^	79.91 ± 1.10^a^	86.37 ± 0.52^b^
K14 2	100^a^	73.11 ± 0.04^c^	69.67 ± 0.57^d^	96.77 ± 0.09^b^	71.62 ± 0.06^c^	57.93 ± 0.75^j^	90.04 ± 2.21^a^
K16	48.65 ± 0.17^h^	65.62 ± 0.23^e^	49.52 ± 0.10^m^	52.73 ± 0.16^h^	54.97 ± 0.09^k^	61.99 ± 0.24^i^	39.63 ± 0.56^i^

*Note*: Values are expressed as averages of three independent experiments ±SD. ND indicates that no biogenic amines have been detected (*p* < .05).

### 3.3 Hemolytic activity of strains

**FIGURE 2:**
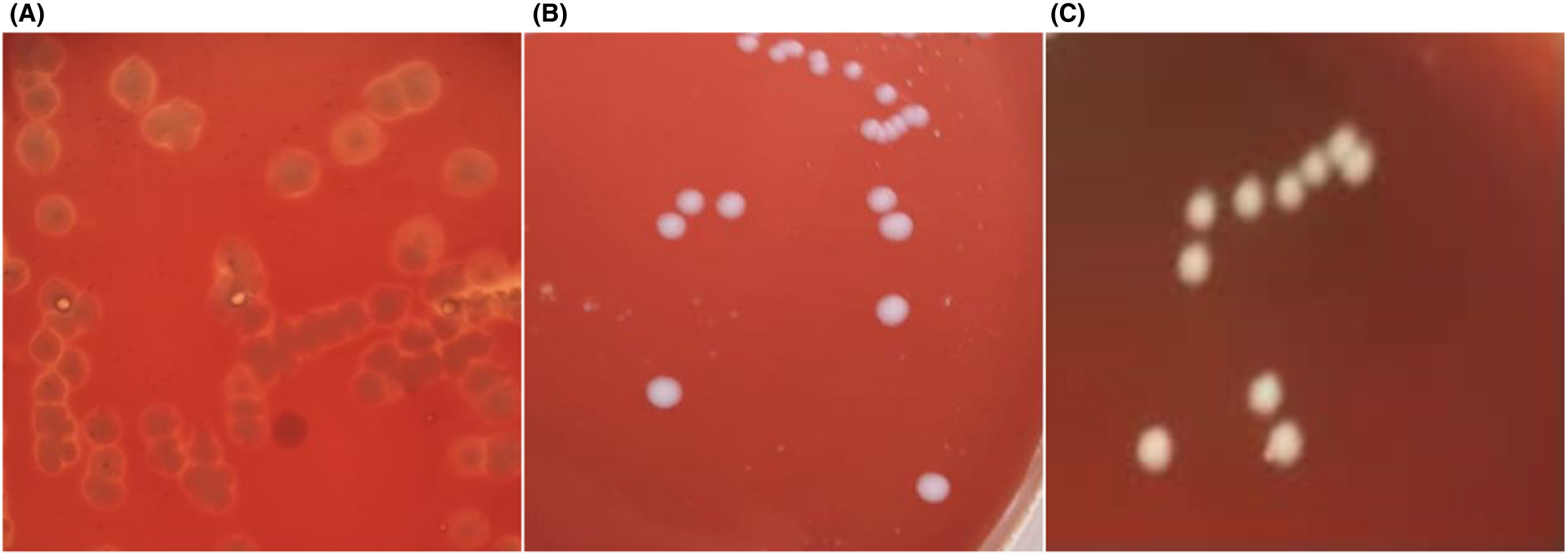
Hemolysis test results of strains. (A) *Listeria monocytogenes*. (B) Lactic acid bacteria. (C) Yeast.

Hemolysis is an important index to evaluate the safety of a bacterial strain (Jeong et al., 2014). Hemolysis activities of the tested LAB and yeast strains were detected. As shown in Figure 2A, there was a transparent aperture around the *Listeria monocytogenes* as the positive control strain, indicating β hemolysis. However, there was no transparent or grass‐green aperture around the LAB and yeast, indicating non‐hemolysis. Jeong et al. (2016) reported that strains isolated from Korea Doenjang were non‐a‐hemolytic, which can remove the potential risk of hemolysis.

### 3.4 Protease enzyme activity of the tested strains

In the fermentation of soybean paste, the protease produced by starter cultures decompose protein in raw materials into smaller molecules, such as peptides and free amino acids, which play a vital role in the flavor and color development of soybean paste (Tang et al., 2020). Therefore, the strains with high protease activity were crucial to improve the quality of soybean paste. Table 6 shows that the protease activities of LAB strains ranged from 71.27 ± 5.14 to 319.00 ± 5.79 U/L, among which the protease activities of strains D43, O10, and M44 were significantly higher than those of other strains. The protease activities of yeast ranged from 4.46 ± 0.64 to 343.55 ± 1.93 U/L, and the protease activities of K13, O2‐6 and M4‐1 were significantly higher than those of other yeast (*p* < .05). Huang (2019) determined the protease enzyme activities of *L. plantarum*, *Lactobacillus rhamnosus*, *Lactobacillus bulgaricus*, and *Lactobacillus casei*, respectively, and the protease enzyme activities were measured as 6.28, 8.98, 6.08, and 47.59 U/mL, respectively. Gong (2015) measured the protease enzyme activity of six strains of *Lactobacillus bulgaricus* after 48 h culture, and the results showed that the enzyme activity of the experimental strains was significantly different, and the enzyme activity of the strains was 15.52, 2.87, 4.12, and 6.77 U/mL in order from low to high. 6.95 and 14.45 U/mL. Although the protease activity of lactic acid bacteria and yeast isolated from soybean paste was lower than that of other strains in the literature, D43, O10, M44, K13, O2‐6, and M4‐1 had the highest enzyme activity among 16 LAB and 15 yeast isolated from soybean paste, respectively. Therefore, the safety of these strains was continued to be evaluated in the follow‐up experiments.

**TABLE 6:** Determination of protease activity of lactic acid bacteria and yeast.

Determination of protease activity of lactic acid bacteria			
Name of strain	Protease activity (U/L)	Name of strain	Protease activity (U/L)
K9	71.27 ± 5.14^j^	D43	319.00 ± 5.79^a^
K3	152.64 ± 9.64^g^	O10	269.46 ± 3.86^b^
K17	202.18 ± 10.29^e^	K16	121.27 ± 2.57^h^
K14	94.00 ± 9.00^i^	M43	124.46 ± 1.93^h^
M16	114.46 ± 3.21^h^	K4	154.46 ± 4.50^g^
WZ	150.36 ± 10.29^g^	M2‐2	174.46 ± 7.07^f^
D2‐6	119.91 ± 1.93^h^	K14 2	234.00 ± 2.57^d^
K6	222.64 ± 7.07^d^	M44	252.18 ± 0.00^c^

Determination of protease activity of yeast			
Name of strain	Protease activity (U/L)	Name of strain	Protease activity (U/L)
K1	119.46 ± 10.29^d^	M2	4.46 ± 0.64^j^
K13	343.55 ± 1.93^a^	M5	20.82 ± 4.50^hi^
K11	29.46 ± 2.57^gh^	K8	5.82 ± 1.29^j^
O2‐6	274.91 ± 10.29^b^	K5	39.00 ± 7.07^g^
M4‐1	204.46 ± 8.36^c^	O2‐1	28.09 ± 5.79^gh^
D4‐3	13.55 ± 3.21^ij^	K10	30.36 ± 5.14^gh^
M1	59.91 ± 5.79^f^	M4‐2	80.36 ± 1.29^e^
D4‐1	69.91 ± 1.93^ef^	‐	‐

*Note*: Values are expressed as averages of three independent experiments ±SD (*p* < .05).

### 3.5 Antibiotic resistance of the tested strains

Antibiotic resistance was related to the safety of strains. Resistant strains can transfer resistant genes into pathogens, so strains for food fermentation should be sensitive to antibiotics (Meng et al., 2021). According to the biogenic amines production and protease activity of the tested strains, six strains were selected for antibiotic resistance assessment. Three strains of LAB showed sensitivity to conventional antibiotics except for vancomycin, as shown in Table 7. D43 and K14 2 were sensitive to chloramphenicol, clindamycin, erythromycin, and ampicillin, while K9 was moderately sensitive to ampicillin and erythromycin. D43 and K14 2 showed moderate susceptibility to kanamycin, while K9 showed resistance to kanamycin. Three strains were resistant to vancomycin. It was reported that *L. plantarum* isolated from Brazilian cocoa were also resistant to vancomycin. Generally, LAB has general resistance to vancomycin (Santos et al., 2016).

**TABLE 7:** Sensitivity of lactic acid bacteria to antibiotics.

Antibiotics	D43		K9		K14 2	
	Inhibition zone diameter (mm)	Antibiotic resistance	Inhibition zone diameter (mm)	Antibiotic resistance	Inhibition zone diameter (mm)	Antibiotic resistance
Chloromycetin	35.28 ± 0.40	S	26.18 ± 0.82	S	33.64 ± 1.64	S
Clindamycin	20.64 ± 1.53	S	30.22 ± 0.54	S	43.84 ± 0.96	S
Erythromycin	33.44 ± 0.79	S	23.54 ± 1.61	S	31.20 ± 0.51	S
Ampicillin	26.76 ± 1.19	S	18.98 ± 1.16	I	25.24 ± 1.07	S
Tetracycline	24.40 ± 1.36	S	12.68 ± 1.30	I	16.26 ± 0.14	I
Gentamicin	19.56 ± 1.36	I	18.40 ± 0.85	I	20.48 ± 0.11	S
Levofloxacin	15.28 ± 0.74	I	13.26 ± 0.82	I	15.70 ± 0.42	I
Kanamycin	19.22 ± 1.16	I	9.98 ± 1.10	R	14.40 ± 0.91	I
Vancomycin	ND	R	ND	R	ND	R

*Note*: Antibiotic resistance: S, sensitive; I, intermediate sensitive; R, resistant.

The antibiotic resistance of yeasts is shown in Table 8, and the three strains showed sensitivity to conventional antibiotics. O2‐6 was susceptible to fluconazole, while M4‐1 and K13 were resistant to fluconazole. O2‐6 was safer than the other two strains. Pilar et al. (2018) reported that yeasts isolated from food showed resistance to some antibiotics.

**TABLE 8:** Sensitivity of yeast to antibiotics.

Antibiotics	M4‐1		O2‐6		K13	
	Inhibition zone diameter (mm)	Antibiotic resistance	Inhibition zone diameter (mm)	Antibiotic resistance	Inhibition zone diameter (mm)	Antibiotic resistance
Econazole	25.73 ± 1.13	S	24.79 ± 1.57	S	15.78 ± 2.08	I
Itraconazole	19.43 ± 1.56	I	22.44 ± 1.49	S	20.79 ± 1.59	S
clotrimazole	30.04 ± 0.96	S	26.59 ± 0.42	S	33.12 ± 0.84	S
ketoconazole	19.45 ± 0.60	I	18.45 ± 0.55	I	21.81 ± 2.04	S
Miconazole	13.10 ± 0.99	I	18.07 ± 1.01	I	15.08 ± 0.98	I
amphotericin	9.47 ± 0.37	R	11.09 ± 0.47	I	10.69 ± 0.10	I
Fluconazole	ND	R	19.28 ± 0.52	I	ND	R

*Note*: Antibiotic resistance: S, sensitive; I, intermediate sensitive; R, resistant.

Abbreviation: ND, not detected.

### 3.6 Results of 16srDNA sequence analysis of strains

According to the results above, the lactic acid bacteria D43 and yeast O2‐6 were considered safety strains and had higher protease activity.

The strain D43 was identified by 16S rRNA sequencing as *L. plantarum* and named *L. plantarum* DPUL‐J8. And yeast O2‐6 was identified by 26S rRNA sequencing as *P. kudriavzevii* and named as *P. kudriavzevii* DPUY‐J8. The phylogenetic trees are shown in Figure S1.

### 3.7 Biogenic amines and aflatoxin B1 levels of soybean paste fermented with selected strains

In this study, soybean paste was made using *Aspergillus niger* DPUM‐J2, *L. plantarum* DPUL‐J8 and *P. kudriavzevii* DPUY‐J8, as shown in Figure 1. The soybean paste samples of four groups fermented with different strains were assessed for biogenic amines, aflatoxin B1, and other physicochemical parameters.

**FIGURE 3:**
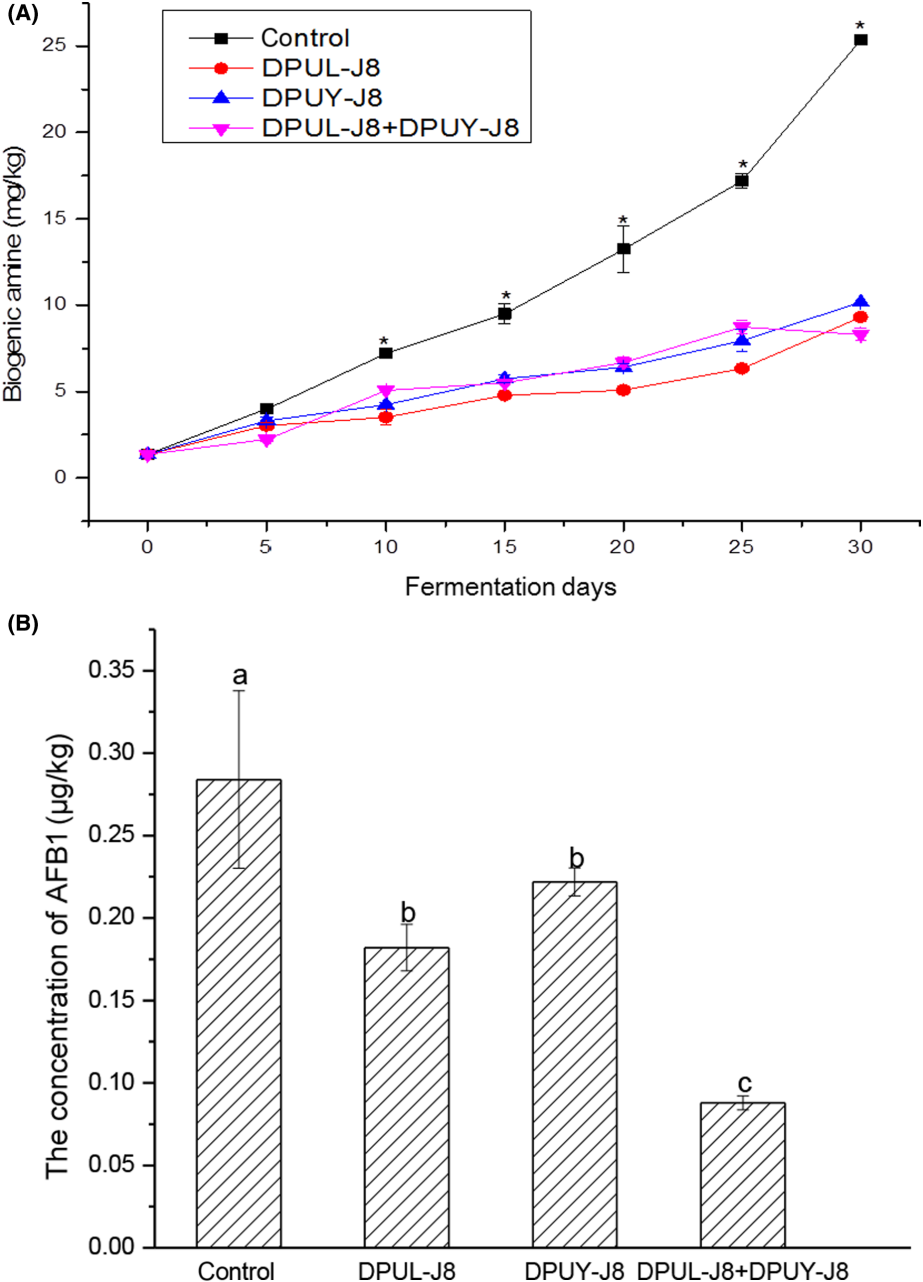
(A) Changes in BAs during soybean paste fermentation. (B) The content of aflatoxin B1 in soybean paste (*p* < .05). Lines with solid square marks (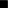) represent blank control; lines with solid up triangle marks (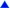) represent inoculated with *Pichia kudriavzevii* DPUY‐J8 (DPUY‐J8); lines with solid circle marks (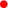) represent inoculated with *Lactiplantibacillus plantarum* DPUL‐J8 (DPUL‐J8); lines with solid downward triangle marks (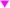) represent co‐culture with *L. plantarum* DPUL‐J8 and *P. kudriavzevii* DPUY‐J8 (DPUL‐J8+DPUY‐J8).

As shown in Figure 3A, the content of biogenic amine gradually increased with the progress of fermentation. While the BAs concentration of soybean paste samples inoculated with *L. plantarum* DPUL‐J8 and *P. kudriavzevii* DPUY‐J8 was obviously lower than that of the control group fermented by *Aspergillus niger* DPUM‐J2 (*p* < .05). The BAs concentration of soybean paste samples inoculated with DPUL‐J8 (*L. plantarum* DPUL‐J8), DPUY‐J8 (*P. kudriavzevii* DPUY‐J8), and DPUL‐J8+DPUY‐J8 (*L. plantarum* DPUL‐J8 and *P. kudriavzevii* DPUY‐J8 together) reached to 9.31 ± 0.14, 10.17 ± 0.07, and 8.31 ± 0.3 mg/kg, respectively, reducing by 62.01%, 63.20%, and 67.15% compared with that of control. Zhao et al. (2020) reported that the combination of *P. acidilactici* M28 and *S. carnosus* as starter cultures was more effective to reduce the accumulation of biogenic amines in soybean paste. Lee et al. (2016) found that using *L. plantarum* D‐103 as starter cultures in miso reduced the histamine content by 58%. We deduced that *L. plantarum* DPUL‐J8 and *P. kudriavzevii* DPUY‐J8 could inhibit BAs production during soybean paste fermentation.

As shown in Figure 3B, the content of aflatoxin B1 in soybean paste samples ranged from 0.08 ± 0.01 to 0.28 ± 0.05 μg/kg, which was lower than 5 μg/kg for aflatoxin B1 in soy sauce and brewing sauce required in Chinese national standard GB2761‐2017, indicating that the aflatoxin B1 content in the fermented soybean paste was at the safe level. The content of aflatoxin B1 in the control group was higher than that in the DPUL‐J8, DPUY‐J8, and DPUL‐J8+DPUY‐J8 groups. Involvement of *L. plantarum* DPUL‐J8 and *P. kudriavzevii* DPUY‐J8 in soybean paste fermentation led to the reduction of aflatoxin content in the soybean paste samples. It was possible that the aflatoxin structure was destroyed and degraded by LAB and yeast.

### 3.8 Physicochemical parameters of soybean paste fermented

The physicochemical parameters, including pH, total acid, reducing sugar, and amino acid nitrogen concentration during the fermentation of four groups of soybean paste were determined.

**FIGURE 4:**
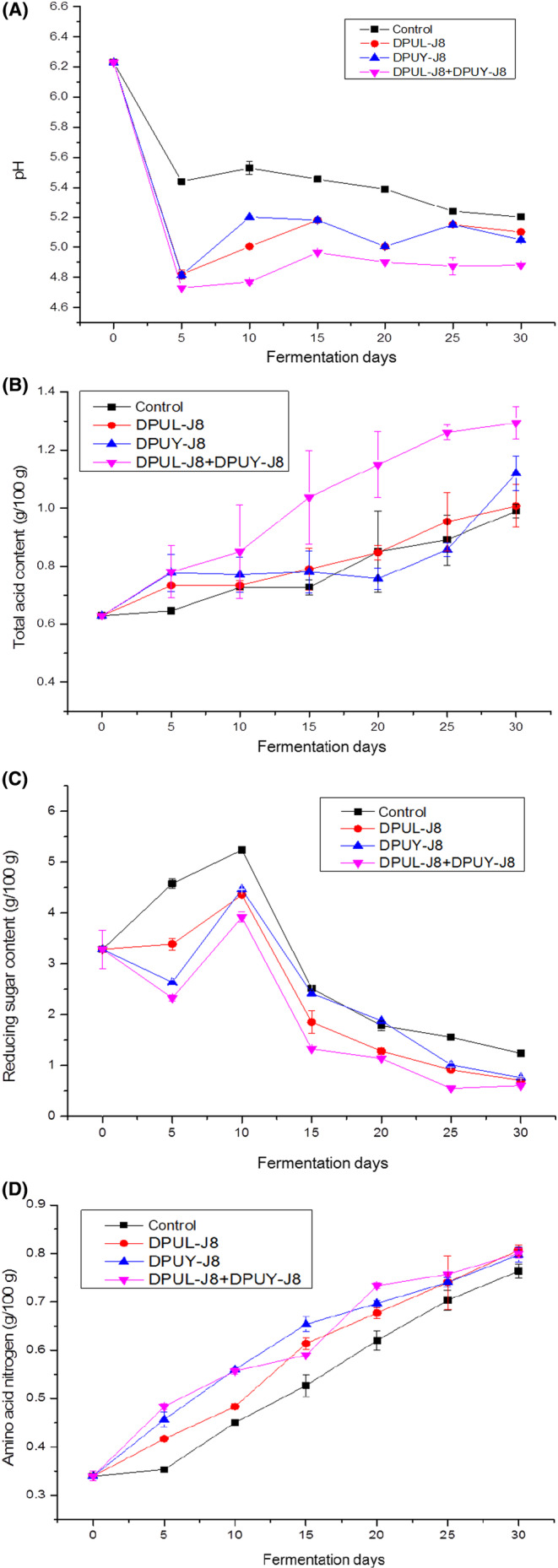
Changes in pH (A), Total acid (B), Reducing sugar (C) and Amino acid nitrogen (D) during soybean paste fermentation. Lines with solid square marks represent inoculated with blank (control); lines with solid up triangle marks represent inoculated with *Pichia kudriavzevii* DPUY‐J8 (DPUY‐J8); lines with solid circle marks represent inoculated with *Lactiplantibacillus plantarum* DPUL‐J8 (DPUL‐J8); lines with solid downward triangle marks represent co‐culture with *L. plantarum* DPUL‐J8 and *P. kudriavzevii* DPUY‐J8 (DPUL‐J8+DPUY‐J8).

The pH value is an essential index during the soybean paste fermentation. As shown in Figure 4A, during the fermentation period, pH values in the soybean paste samples decreased sharply and then remained steady. The pH values of samples DPUL‐J8, DPUY‐J8, and DPUL‐J8+DPUY‐J8 maintained around 5.05 ± 0.03, 5.10 ± 0.02, and 4.88 ± 0.03 after the fermentation was completed, respectively, which were lower than that of the control. Microorganisms might decompose carbohydrates and fats during the soybean paste fermentation to produce small molecular organic acids such as lactic acid and acetic acid.

As for total acid, its concentration increased significantly during the whole soybean paste fermentation with values ranging from 0.63 ± 0.01 g/100 g to 1.29 ± 0.05 g/100 g (Figure 4B), which conform to the Chinese national standard GBT20560‐2006. The content of total acid in DPUL‐J8+DPUY‐J8 was slightly higher than that in control, which was commensurate with pH value. Acid substances were produced due to the mass reproduction and metabolism of LAB and yeast in the soybean paste, resulting in an increase in the total acid content. The total acid content in the soybean paste can be used to indicate the number of acid‐producing microorganisms during fermentation. On the other hand, lactic acid and succinic acid were the dominant organic acids in the soybean paste. Organic acids were also the raw materials for producing flavor substances such as esters.

The content of reducing sugar is closely related to the flavor in soybean paste and has a critical effect on the formation of color and flavor of soybean paste, which is an important parameter reflecting the quality of soybean paste. The change in reducing sugar during the soybean paste fermentation is shown in Figure 4C. The concentration of reducing sugar increased initially and decreased afterward. The result was in accordance with the result of Cui et al. (2015). Starch in soybean raw materials was hydrolyzed to produce reducing sugar in the first 10 days. With the fermentation, many microorganisms grew up and reproduced, and the reducing sugar was consumed as a carbon source. The reducing sugar content of DPUL‐J8, DPUY‐J8, and DPUL‐J8+DPUY‐J8 was lower than that of the control group sample after the fermentation was completed, indicating that the reducing sugar content was reduced due to the consumption of LAB and yeast during mass propagation. Reducing sugar reflex with amino to produce the Maillard reaction so that the color of the soybean paste was deepened, and the content of the reducing sugar was closely related to the flavor substances of the soybean paste.

Amino acid nitrogen exists in proteins and polypeptides compounds and can release various amino acids after degradation. During the soybean paste fermentation, the protein is used as the raw material, which could be degraded into polypeptide, oligopeptides, and amino acids. Most of the amino acids in soybean paste are valuable flavor compounds and nutrients. Therefore, the concentration of amino acid nitrogen is generally considered the crucial index of soybean paste. The change in amino acid nitrogen content is shown in Figure 4D. The content of amino acid nitrogen ranged from 0.34 ± 0.01 g/100 g to 0.80 ± 0.01 g/100 g, and it conformed to the Chinese national standard GB/T24399‐2009 that the content of amino acid nitrogen in soybean paste should not be less than 0.5 g/100 g. With the prolongation of fermentation days, the content of amino acid nitrogen was continuously increased, because the protease degraded protein in soybean raw materials into peptides and amino acids during the fermentation, and the accumulation of amino acids increased the concentration of amino acid nitrogen. During the soybean paste fermentation, the content of amino nitrogen in the treatment samples was higher than that in the control group, which was due to the decomposition of protease secreted by LAB and yeast to produce amino acids, which increased the content of amino nitrogen in the soybean paste samples.

### 3.9 Analysis of volatile flavor compounds in soybean paste samples

The HS‐SPME and GC–MS technologies were used to detect the types and contents of volatile substances in the four groups of soybean paste. As shown in Table 9, a total of 26 kinds of volatile compounds were detected in soybean paste from different groups, including four types of alcohols, three types of aldehydes, four types of esters, three types of ketones, four types of acid phenols, three types of pyrazines and others. The soybean paste samples inoculated with *L. plantarum* DPUL‐J8 and *P. kudriavzevii* DPUY‐J8 exhibited more volatile compounds.

**TABLE 9:** Concentration of volatile flavor compounds in soybean paste (μg/g).

Compounds	RI	Control	DPUL‐J8	DPUY‐J8	DPUL‐J8+DPUY‐J8
Phenethyl alcohol	1916	0.28 ± 0.07^e^	25.33 ± 5.93^a^	15.13 ± 3.24^a^	112.26 ± 25.81^a^
1‐Octen‐3‐ol	1438	‐	2.38 ± 0.59^e^	‐	1.40 ± 0.26^k^
2‐Hexen‐1‐ol	992	‐	‐	0.21 ± 0.06^h^	‐
2‐Hexadecanol	1702	‐	‐	‐	1.80 ± 0.52^j^
Benzaldehyde	1530	6.20 ± 1.78^a^	2.72 ± 0.81^d^	2.15 ± 0.71^e^	0.70 ± 0.12^m^
Phenylacetaldehyde	1642	2.69 ± 0.22^c^	12.88 ± 3.23^b^	15.18 ± 2.91^a^	3.15 ± 0.76^h^
Nonanal	1395	‐	‐	‐	0.51 ± 0.15^m^
Ethyl palmitate	1993	1.10 ± 0.20^d^	1.12 ± 0.13^f^	1.03 ± 0.23^f^	4.44 ± 1.21^g^
Octadecadienoic acid ethyl ester	2162	1.00 ± 0.02^d^	0.88 ± 0.24^h^	1.02 ± 0.31^f^	5.19 ± 1.76^f^
Ethyl oleate	2173	0.12 ± 0.03^g^	0.70 ± 0.17^i^	1.07 ± 0.13^f^	2.76 ± 0.60^i^
Germacra	1128	‐	‐	3.80 ± 0.87^d^	1.14 ± 0.31^L^
2‐Hydroxy‐5‐methyl acetophenone	1316	‐	‐	‐	8.00 ± 2.36^d^
Palmitic acid	1968	‐	‐	‐	4.49 ± 1.35^f^
4‐Hydroxy‐3‐methoxystyrene	1317	‐	‐	‐	4.51 ± 1.12^f^
2‐methoxy‐4‐phenol	1450	‐	0.99 ± 0.28^g^	‐	7.08 ± 1.09^e^
2,6‐Ditertbutyl‐4‐methylphenol	1513	‐	‐	0.18 ± 0.11^i^	0.19 ± 0.01^m^
Dimethyl pyrazine	1332	2.86 ± 0.71^b^	11.66 ± 2.91^c^	8.19 ± 1.92^c^	4.71 ± 0.98^f^
Trimethyl pyrazine	1409	‐	‐	13.21 ± 3.28^b^	‐
Tetramethyl pyrazine	1089	‐	‐	0.25 ± 0.04^h^	31.65 ± 5.39^b^
Dimethoxy benzene	1369	‐	‐	0.56 ± 0.13^f^	‐
Tetradecane	1400	0.19 ± 0.04^f^	0.33 ± 0.12^j^	0.34 ± 0.11^gh^	0.88 ± 0.25^m^
Hexadecane	1600	‐	0.35 ± 0.14^j^	0.40 ± 0.06^g^	0.94 ± 0.16^m^
Nonadecane	1900	‐	‐	0.10 ± 0.02^i^	‐
3‐ethyl‐5‐methyl‐Benzne	1238	‐	‐	‐	11.87 ± 3.04^c^

*Note*: Values are expressed as averages of three independent experiments ±SD (*p* < .05). “‐” indicates that no detected.

Alcoholic compounds can give the soybean paste a pleasant unique aroma. Enol, phenylethanol, etc., are usually produced by the amino acid metabolism of yeast in soybean paste. Phenethyl alcohol was detected in every group of soybean paste samples, and the maximum value in the DPUL‐J8+DPUY‐J8 group was 112.26 μg/g, respectively, which was much higher than other groups and gave the soybean paste a strong floral aroma. Moreover, the soybean paste fermented by different strains also produced individually characteristic alcohol compounds. For example, 1‐Octen‐3‐ol was detected only in DPUL‐8 and DPUL‐J8+DPUY‐J8, while 2‐Hexen‐1‐ol was only present in DPUY‐J8. The alcohols were possibly produced by yeast and the metabolism of amino acids (Liu et al., 2020). Ester compounds can impart a pleasing ester flavor to the soybean paste. The main volatile compounds in the fermented soybean paste were esters (Lee et al., 2013). Ethyl linoleate, and ethyl oleate were the esters co‐detected in four samples, the content of ethyl linoleate (5.19 μg/g) and ethyl oleate (2.76 μg/g) in the soybean paste inoculated with *L. plantarum* DPUL‐J8 and *P. kudriavzevii* DPUY‐J8 was 1–5 times higher than that of the control group. This may be because of the esterification reaction between alcohols with organic acids under the enzymes produced by LAB and yeast (Liu et al., 2019). The formation of esters was strongly associated with protease and esterase activities, which increased as fermentation proceeded (Kum et al., 2015). We found that acids were only detected in the sample DPUL‐J8+DPUY‐J8, with the palmitic acid concentration reaching 4.49 ± 1.35 μg/g. Organic acids were generated by the fermentation of microorganisms such as lactic acid bacteria or the decomposition reaction of esters in soybean paste fermented grains (Jeong et al., 2013). 2‐Hydroxy‐5‐methyl acetophenone with a pungent flavor was detected in soybean paste of the DPUL‐J8+DPUY‐J8 group, and it contributed significantly to the aroma components of soybean paste. Ketones are generally produced through three pathways: microbial metabolism, thermal oxidative degradation of unsaturated fatty acids, and amino acid degradation (Lee & Ahn, 2009). Phenylacetaldehyde and benzaldehyde, which were generated by the oxidation of higher alcohols and unsaturated fatty acids, were detected in all groups of soybean paste samples. Furthermore, pyrazines also significantly affected the flavor of soybean paste. More pyrazines in the DPUY‐J8 and DPUL‐J8+DPUY‐J8 groups were detected, contributed greatly to the potato flavor of soybean paste (Liu et al., 2019). Therefore, yeast and LAB have a great contribution to the flavor and taste of the soybean paste.

## 4 CONCLUSION

In this study, *L. plantarum* DPUL‐J8 and *P. kudriavzevii* DPUY‐J8 were proved to be safe strains by hemolysis and antibiotic resistance. The application of these two strains in the soybean paste fermentation could effectively inhibit the formation of biogenic amines, aflatoxin B1, and other harmful substances, improve the physical and chemical indices of soybean paste, and produce more desirable flavor compounds than the control. Therefore, using *L. plantarum* DPUL‐J8 and *P. kudriavzevii* DPUY‐J8 as the starter cultures in the soybean paste fermentation might be a good strategy to reduce the formation of harmful substances and improve the general quality during soybean paste production.

## AUTHOR CONTRIBUTIONS

**Siyi Li:** Data curation (equal); investigation (equal); methodology (equal); writing – original draft (equal); writing – review and editing (equal); visualization (equal). **Linjie Guo:** Data curation (equal); investigation (equal); methodology (equal); writing – original draft (equal); writing – review and editing (equal). **Jinhong Gu:** Methodology (equal); software (equal). **Guangqing Mu:** Supervision (equal); writing – review and editing (equal); funding acquisition (equal). **Yanfeng Tuo:** Supervision (equal); writing – review and editing (equal); funding acquisition (equal). All authors have read and agreed to the published version of the manuscript.

## CONFLICT OF INTEREST STATEMENT

The authors declare no conflict of interest.

## Supporting information


Figure S1

## Data Availability

The authors will supply the relevant data in response to reasonable requests.
