# Supplementary material for: Screening lactic acid bacteria and yeast strains for soybean paste fermentation in northeast of China
Source: Food Sci Nutr. 2023 Jul 19;11(8):4502–15. doi: 10.1002/fsn3.3372 (PMC10420762; doi:10.1002/fsn3.3372)
Supplement: Supplementary file 1 — Figure S1 [file FSN3-11-4502-s001.docx]

DPUL-J8





DPUY-J8

(Figure S1)
